# Production of Functional Soluble Dectin-1 Glycoprotein Using an IRES-Linked Destabilized-Dihydrofolate Reductase Expression Vector

**DOI:** 10.1371/journal.pone.0052785

**Published:** 2012-12-26

**Authors:** Say Kong Ng, Tessa Rui Min Tan, Yang Wang, Daniel Ng, Lin-Tang Goh, Muriel Bardor, Victor Vai Tak Wong, Kong Peng Lam

**Affiliations:** 1 Bioprocessing Technology Institute, Agency for Science, Technology and Research (A*STAR), Singapore, Singapore; 2 Department of Pharmacy, Faculty of Science, National University of Singapore, Singapore, Singapore; Institute of Molecular and Cell Biology, Singapore

## Abstract

Dectin-1 (CLEC7A) is a C-type lectin receptor that binds to β-glucans found in fungal cell walls to act as a major pattern recognition receptor (PRR). Since β-glucans epitope is not present in human cells, we are of the opinion that Dectin-1 can have therapeutic functions against fungal infections. We thus set out to produce a soluble extracellular domain of murine Dectin-1 (called sDectin-1) in sufficient titers to facilitate such studies in mouse models. Since sDectin-1 has previously been shown to be glycosylated, we chose to produce this protein using Chinese Hamster Ovary (CHO) cells, a mammalian host cell line suitable for the high-titer production of recombinant glycoproteins. To ensure a high titer production of sDectin-1 and minimize the effects of gene fragmentation, we constructed a mammalian expression vector with a PEST-destabilized dhfr amplifiable marker downstream of an attenuated IRES element, which was in turn downstream of the sDectin-1 gene and a CMV IE promoter. Stably transfected and MTX-amplified cell pools were generated using this vector, and maximum sDectin-1 titers of 246 mg/l and 598 mg/l were obtained in shake flask batch culture and bioreactor fed-batch culture respectively. The purified recombinant sDectin-1 was shown to be glycosylated. Protein functionality was also demonstrated by its ability to bind to zymosan particles and to the cell wall of *Saccharomyces cerevisiae*. We describe for the first time the use of an attenuated IRES-linked PEST-destabilized dhfr amplifiable marker for the production of recombinant proteins with stably amplified cell pools. With our process, we reached the highest reported titer for producing recombinant proteins smaller than 50 kDa in cell pools. sDectin-1 protein produced is glycosylated and functional. This vector design can thus be used efficiently for the high-titer production of functional recombinant proteins.

## Introduction

Dectin-1, also known as CLEC7A, is a type II transmembrane C-type lectin receptor that recognizes β-glucans through its single C-type lectin-like domain at its extracellular region [1]–[3]. This recognition is known to be independent of Ca^2+^ ions, and is responsible for the receptor's role as a major pattern recognition receptor (PRR) by binding to cell walls of different fungal species, such as *Saccharomyces cerevisiae*, *Candida albicans*, *Pneumocystis carinii*, *Coccidioides posadasii*, and *Aspergillus fumigatus* [4]–[7]. Binding to particulate but not soluble β-glucans triggers intracellular signaling through Dectin-1's immunoreceptor tyrosine-based activation motif (ITAM)-like motif in its cytoplasmic tail to result in cell responses, including phagocytosis, respiratory burst, and production of various cytokines and chemokines (e.g. TNF, CXCL2, IL-2, IL-10, IL-12) [4]–[5], [8]–[11]. Dectin-1 has been shown to be expressed in many cell types, including dendritic cells, macrophages, monocytes and neutrophils [12]. Expression of Dectin-1 is high at the lungs, guts, thymus and spleen, consistent with its role as a surveillance receptor for pathogens [13] and its essential role in the control of fungal infections [14], [15].

In addition to immune responses against fungal infections, Dectin-1 has been implicated in the induction of autoimmune arthritis. Indeed, Dectin-1 stimulation triggered the development of autoimmune arthritis in genetically prone SKG mice reared in specific pathogen-free (SPF) environment. This signaling is blocked with an antagonistic antibody against Dectin-1 which delayed the onset of disease development [16]. In humans, Dectin-1 mRNA and protein expression were also found to be high in synovial tissue of rheumatoid arthritis patients [17].

Even though Dectin-1 has important physiological functions, there is only one publication to-date that describes an application of soluble Dectin-1 (sDectin-1, modified protein containing the extracellular domain of Dectin-1) as a reagent to detect β-glucans [18]. As β-glucans are not produced by human cells, we opined that the specific binding of Dectin-1 to this epitope [19] can have therapeutic functions, such as pathogen separation from blood [20], delaying pathogen-stimulated arthritis [16] and as a broad spectrum drug delivery agent [21]. To investigate such possibilities in mouse models, we aim to produce murine sDectin-1 in sufficient titers.

sDectin-1 has already been produced as chimeric proteins to various tags using *Escherichia coli*, COS-1, HEK293 cells as well as an *E. coli* cell-free translation system, for studies on the structure and functions of Dectin-1 [1], [18]–[19], [22]–[23]. Human Dectin-1 extracellular domain (ECD) from amino acids 68 or 71 to 247 and murine Dectin-1 ECD from amino acids 67, 69 or 73 to 244 were incorporated into sDectin-1. Production titers were not mentioned for these publications with one exception where up to 8 mg of murine sDectin-1 was recovered from 1.5 L of *E. coli* culture. Nonetheless, the recombinant protein titers from these studies are expected to be low, because no cell line or process optimization was performed. Since sDectin-1 from mammalian cells was shown to be glycosylated and interacted with T-cells to a lesser extent to that from *E. coli* [1], an optimized protein production process using a mammalian cell system will be desirable for the development for therapeutic applications.

To achieve a high-titer production of recombinant proteins, the mammalian cell expression system popularly used in the biopharmaceutical industry is the methotrexate (MTX) amplification system [24]–[26]. In this system, MTX is applied to cells co-transfected with genes encoding the recombinant protein and the dihydrofolate reductase (dhfr) enzyme. As MTX is an inhibitor of dhfr, increasing MTX concentrations results in an increase in gene copy number of dhfr, and the collocated recombinant protein [27]. Dhfr deficient Chinese hamster ovary (CHO) cells, such as the CHO-DG44 cell line [28], are commonly used in this system since they lack functional endogenous dhfr gene which may be amplified when MTX is applied.

Technologies to increase the efficiency and effectiveness of such amplification systems by shortening the time required to isolate high-producing cell lines and increasing expression levels of recombinant genes were recently reviewed [29], [30]. These technologies include modifying the expression of selection marker to improve drug selection, using DNA elements or promoters that allow higher gene expression, optimizing the codon usage of the product gene, inserting recombinant genes into genomic sites of high expression by gene targeting, and cell engineering to modify cell growth, cell metabolism, cell death and protein production.

Amongst these technologies, expression vector modifications remain the most convenient and easily implementable for new cell line development projects, especially for laboratories without pre-engineered or pre-targeted host cell lines. To this effect, we have previously demonstrated that destabilizing sequences can be applied on dhfr selection marker to improve the productivity of cell lines producing recombinant human interferon gamma (hIFNγ) [31]. In this study, the selection marker was driven by the weak Herpes simplex virus thymidine kinase (HSV-tk) promoter and further destabilized using the murine ornithine decarboxylase (MODC) PEST for protein degradation [32] and the AU-rich elements (ARE) for mRNA degradation [33]. This destabilized selection marker was then adjoined with the hIFNγ gene driven by the strong human cytomegalovirus (hCMV) promoter on a dicistronic vector. This resulted in high selection stringencies, and corresponding titer and specific productivity improvements of 12.6 and 13.3 folds respectively in stably transfected cell pools as compared to the non-destabilized vector. These cells can also be MTX amplified to further increase hIFNγ product titers.

In the above study, a possible issue with this dicistronic vector design was highlighted when MTX amplification was not successful for one cell pool. One possible reason for this phenomenon is gene fragmentation. If the selection marker gene and the gene-of-interest were to be separated in some cells during the transfection process, MTX amplification may not successfully improve the recombinant protein titer in these cells, since the amplified DNA will be a 100 to 3000 kilobase region around the dhfr gene [34], [35]. To verify this, we have previously demonstrated that gene fragmentation can occur at a significant level of 14% during stable transfection of CHO-DG44 cells [36]. Due to this phenomenon, time-consuming cloning and screening steps are often necessary in cell line development processes to find high producing clones.

A closer association of the selection marker to the gene-of-interest through the use of an Internal Ribosome Entry Site (IRES) element may be a potential solution to this problem, as this approach has been reported to prevent rearrangement or deletion of genes during MTX amplification [37] and to ensure successful selection of recombinant protein producing cells using other selection markers [38]–[40]. With this approach, both the gene-of-interest and the selection marker will be transcribed into a single mRNA transcript, and this transcript will in turn be translated into 2 proteins. This will help to improve the co-localization of these two genes for successful MTX amplification. With this vector design, an important implication is that the MTX amplification of stably transfected cell pools is possible, and time-consuming cloning and screening steps may be omitted, while retaining high recombinant protein productivity in the amplified cell pools.

In this report, we describe for the first time, the expression vector design combining the use of destabilized selection marker with an attenuated IRES element to ensure a high titer production of sDectin-1 while mitigating gene fragmentation for successful MTX amplification. The purification, characterization and functionality of sDectin-1 are also reported.

## Results and Discussion

### Design of sDectin-1 mammalian expression vectors

**Figure 1:**
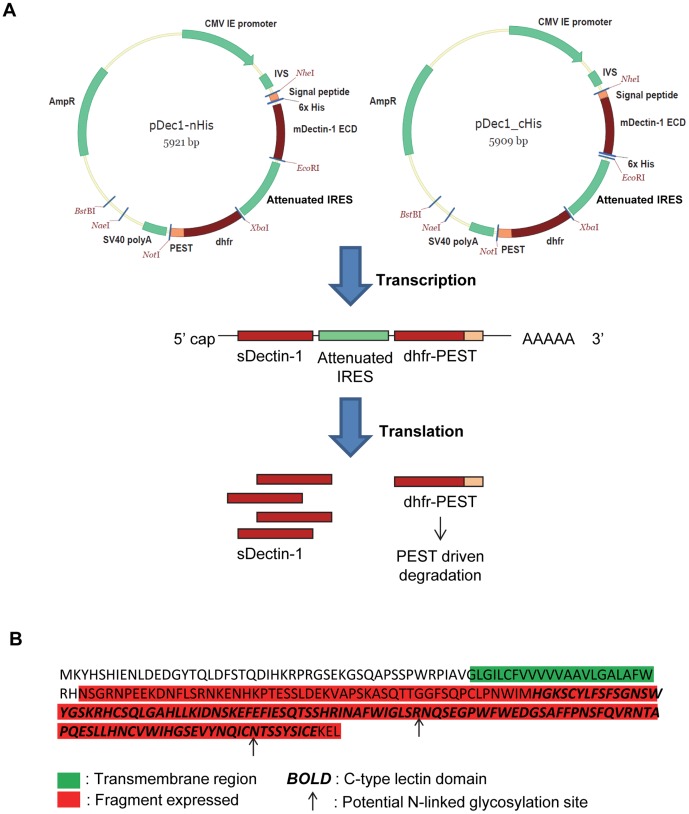
Design of sDectin-1 expression vector. (A) Vector map of pDec1-nHis and pDec1-cHis, and illustration of its design to enhance sDectin-1 production. (B) Amino acid sequence of mDectin-1 from bone-marrow derived macrophage cells of C57BL/6 mice. The peptide fragment expressed in sDectin-1, the transmembrane region, the C-type lectin domain and the two potential N-linked glycosylation sites are indicated on the sequence based on alignment to protein sequence from Uniprot Accession Q6QLQ4.

To limit gene fragmentation for the production of sDectin-1, we associated the destabilized dhfr selection marker to sDectin-1 using an IRES element, for both the sDectin-1 and the selection marker genes to be transcribed into a single mRNA, which will in turn be translated into 2 proteins (**Fig. 1A**). The selection marker gene is also placed downstream of the promoter, sDectin-1 gene and the IRES element, so that its transcription is dependent on the successful transcription of the sDectin-1 gene driven by the promoter upstream. Under selection pressure, this design thus minimizes or prevents the survival of cells with sDectin-1 gene dissociated from the selection marker at the DNA level [37]–[40], which is important for the subsequent MTX amplification step, because colocalization of the sDectin-1 gene and the dhfr gene is essential for amplification to be successful [34], [35].

Since both sDectin-1 and selection marker genes are theoretically transcribed into a single mRNA, improvement of selection stringency by reducing transcript levels with the use of a weak promoter or AU-rich elements (ARE) may likely also reduce the productivity of the sDectin-1 gene. Hence, these were not applied here, in contrast to our previous study [31]. Instead, to maximize the expression of the sDectin-1 gene, we chose to use the cytomegalovirus immediate early promoter (CMV IE promoter) for inducing a constitutive high transcription of the sDectin-1 gene, IRES and dhfr sequences in the same mRNA transcript. With the high transcription of the dhfr gene, selection stringency can only be imposed at the translation and post-translation stages of the dhfr selection marker protein expression. To mitigate translation efficiency, the choice of IRES elements is important: From the many reported IRES elements [41], we decided to use the IRES element from the Encephalomyocarditis virus (EMCV) [42] since it is well-characterized [43], [44]. Of the variants of the EMCV IRES, the attenuated version found in pIRES vector [39] has been reported to produce 8 to 10 fold less protein than the wild-type EMCV IRES [44]. As such, we chose this attenuated IRES element to impose a low translation efficiency of the dhfr selection marker protein. To further improve the selection stringency post-translation, the PEST protein degradation signal [32] is added to result in a higher degradation rate of the dhfr selection marker protein (**Fig. 1A**). The lower translation rate of the attenuated IRES and the higher protein degradation rate of the PEST motif are thus combined to have a reducing effect on the intracellular recombinant dhfr protein level (**Fig. 1A**). This, in turn, will likely result in a higher sDectin-1 expression upon MTX amplification, since the cells will require more gene copies or transcripts to survive the selection with the weakened dhfr expression, as described previously [31].

A mammalian expression vector for recombinant sDectin-1 was therefore constructed as described below. Murine Dectin-1 (mDectin-1) coding sequence was first determined to be identical to NCBI Reference Sequence NP_064392.2 by cloning it from first-strand cDNA of murine bone-marrow derived macrophage cells from C57BL/6 mice. A sequence that is codon-optimized for CHO cells was then synthesized (Genscript, Piscataway, NJ). As mDectin-1 is a Type II transmembrane protein, the extracellular domain to be expressed in sDectin-1 was designed to be amino acid 73 to 244 of the NCBI reference sequence (**Fig. 1B**). A human IgG kappa light chain leader sequence [45] was added upstream of this coding sequence as a signal peptide for the secretion of the sDectin-1. A 6× histidine tag was also added to either the N- or C-terminus of the protein to facilitate protein purification. These coding sequences were inserted into a mammalian expression vector downstream of a CMV IE promoter, and were followed by an attenuated version of the IRES from EMCV [39], and a destabilized dhfr selection marker consisting of a chimeric dhfr-PEST sequence [31] (**Fig. 1A** and **Fig. S1**).

### Development and evaluation of sDectin-1 producing cell pools

After suspension CHO-DG44 cells were transfected with the linearized expression vectors, C- and N-terminal histidine tagged sDectin-1 proteins (cHis-sDectin-1 and nHis-sDectin-1 respectively) were detected in culture supernatants using a polyclonal antibody against mDectin-1. The expression of the sDectin-1 proteins were also confirmed by peptide mass fingerprinting using an LC-MS/MS approach. At least 6 unique peptides corresponding to mDectin-1 were detected with protein coverage of 34%. The analysis also confirmed that the signal peptide was correctly cleaved from the mature secreted protein (Data not shown).

**Figure 2:**
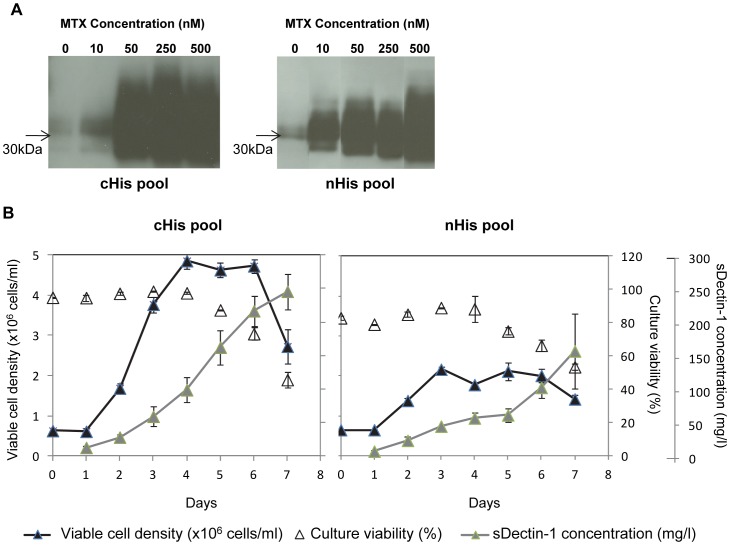
MTX amplification and characterization of sDectin-1 cell pools. (A) Western blotting of supernatant samples from cultures adapted to different MTX concentrations using an mDectin-1 goat polyclonal antibody (1∶1000; AF1756; R&D Systems) with a HRP conjugated anti-goat antibody (1∶2000; Catalog number V8051; Promega). cHis pool and nHis pool are cell pools producing sDectin-1 with histidine tagged at the C- and N-terminals respectively. (B) Shake flask batch culture cell growth and sDectin-1 production profiles of sDectin-1 producing cell pools. Values shown represent mean values obtained from three replicate flasks. Error bars indicate the standard deviation of the experiment.

The transfected CHO cells then underwent selection and MTX amplification in DMEM media containing 1% of fetal bovine serum. The low serum supplementation resulted in the adherence of live cells to tissue culture flasks, allowing an easy separation between live and dead cells. During the MTX amplification process, the expression of sDectin-1 increased in the culture supernatant as detected by western blot (**Fig. 2A**). Cell pools at 500 nM MTX concentration were then adapted to suspension culture. This sDectin-1 cell line development process from transfection to amplified suspension cultures took 17 weeks. In retrospect, this timeline can be further shortened for the cell pool producing cHis-sDectin-1 (cHis pool), since the 250 nM MTX culture seemed to have similar sDectin-1 expression when compared to the 500 nM MTX culture, and the cells took 5 weeks to adapt to 500 nM MTX. In any case, this is a significantly shorter production cell development timeline using MTX amplification, for which a 6 month timeline is typically needed due to the limiting dilution steps which does not occur in our study.

The growth and protein production curves of these suspension cell pools producing cHis- or nHis- sDectin-1 were then determined in shake flask batch cultures (**Fig. 2B**). Both recombinant cell pools exhibited lag phase of 1 day, even though the cultures were seeded at about 0.5×10^6^ cells/ml. Nevertheless, the cHis pool reached a high peak cell density of 4.9±0.2×10^6^ cells/ml, whereas 2.2±0.1×10^6^ cells/ml was attained by the cell pool producing nHis-sDectin-1 (nHis pool). The specific growth rate of the cHis pool during exponential phase was 0.038 h^−1^, 1.5 fold that of the nHis pool (0.025 h^−1^). The removal of selection pressure by the addition of a hypoxanthine and thymidine (HT) supplement or the removal of MTX from the culture medium did not improve the cell growth of nHis pool (Data not shown), suggesting that MTX inhibition was not the direct cause of this lower peak cell density. Since the two cell pools behaved similarly during MTX amplification, we speculate that this difference in cell growth may be dependent on stochastic events during the cell line development process, such as the integration sites of the expression vector into the cell genomes, and the survival of dominant cells during selection and passaging. Despite the difference in peak cell density, culture viabilities of these two cell pools both started to decrease on day 4, and went below 60% on day 7. This suggests that the consumption of limiting nutrients were similar in both cell pools. Corresponding to the observed cell densities, the recombinant sDectin-1 production was higher on day 7 in the cHis pool reaching 246±27 mg/l compared to 158±57 mg/l for the nHis pool. As such, the specific protein productivities at exponential growth phase were similar at 4.3 and 4.9 pcd respectively for the cHis and nHis pools. Considering the whole batch process, the overall specific protein productivities were similar, at 2.6 and 3.0 pcd for the cHis and nHis pools respectively. The similar specific protein productivities suggest that the sDectin-1 genes have amplified to similar extent in these two cell pools. This can be further attributed to our expression vector design in which the sDectin-1 genes were linked to the same destabilized dhfr selection marker via the IRES element. This will result in similar ratios of the genes at both ends of the IRES element as described previously [46], and as such, similar sDectin-1 specific productivities with similar extents of MTX amplification.

### Bioreactor production of sDectin-1

Since the cHis pool gave a higher protein titer, this cell pool was chosen for scale-up using a 2 L stirred tank bioreactor in fed-batch mode to further improve cHis-sDectin-1 productivity as well as to produce cHis-sDectin-1 for functional testing. Cells were seeded into 1.5 L HyQ PF-CHO medium at 0.3×10^6^ cells/ml. Glucose and glutamine were maintained at 0.5 g/l and 0.3 mM respectively by addition of a concentrated DMEM-based protein-free feed. The feed was changed to a 200 g/l glucose solution from day 7 to maintain only the culture glucose concentration.

**Figure 3:**
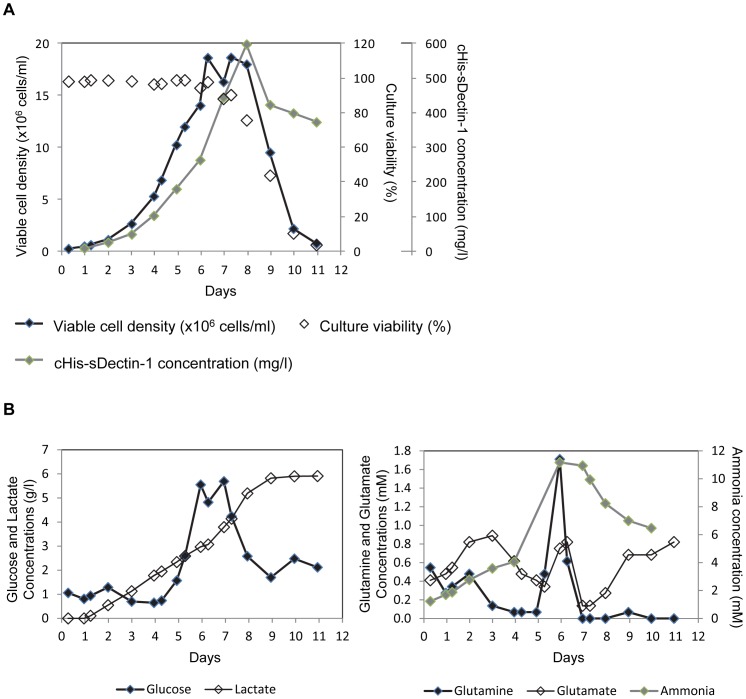
Bioreactor fed-batch production of cHis-sDectin-1 using the cHis cell pool in 500 nM MTX. (A) Cell growth and cHis-sDectin-1 production profiles in 2 L stirred tank bioreactor. (B) Metabolite profiles of the bioreactor culture.

Using this culture mode, viable cell density increased till day 6 to reach 18.6×10^6^ cells/ml (**Fig. 3A**). When compared to the shake flask batch culture (**Fig. 2B**), this is a 2 day extension in cell growth phase and a 3.8 fold improvement in maximum viable cell density. Extension in cell growth phase can be partially attributed to the lower cell seeding density at 0.3×10^6^ cells/ml in the bioreactor fed-batch culture, compared to 0.5×10^6^ cells/ml in the shake flask batch culture. Other than this, the observed cell growth improvement is typical of fed-batch cultures and likely a result of the feeding regime. An accumulation of feed nutrients was noted on day 6 (**Fig. 3B**), likely because our feeding regime assumed a substantial cell growth based on the growth rate determined from the previous sampled time interval, while the culture entered stationary phase in the experiment. Cell viability of the fed-batch culture first dropped below 90% on day 7, which may be due to the depletion in glutamine and ammonia accumulation (**Fig. 3B**). Subsequently, since the feed medium was changed to a 200 g/l glucose solution, glutamine remained depleted and the culture entered the death phase. Although the fed-batch cell growth profile seem superior to that of the batch culture, specific growth rate at exponential growth phase was calculated to be 0.029 h^−1^, 76% of that observed in the batch culture. This lower growth rate may be due to the lower glucose and glutamine concentrations in the medium, since it has been reported that specific growth rate of CHO cells will be decreased with glucose concentrations below 1.22 mM [47] and glutamine concentration below 2 mM [48]. With the lower cell seeding density and specific growth rate in the fed-batch culture, the integral viable cell density (IVCD) was consistently lower than that of the batch culture when the same time points were compared, despite the higher peak cell density achieved: On day 7, IVCD of the fed-batch culture was 44.0×10^9^ cells.day/l, 41% that of the batch culture at 106.3×10^9^ cells.day/l.

Looking at cHis-sDectin-1 production, its titer increased till day 8 despite the glutamine depletion and lack of further cell growth from day 7, suggesting that the production of cHis-sDectin-1 was not growth associated. Furthermore, even though the specific cell growth and IVCD were lower in the fed-batch culture compared to the batch shake flask culture, the recombinant cHis-sDectin-1 protein titer reached a peak of 598 mg/l on day 8 in the fed-batch culture, 2.4 fold higher than the day 7 peak protein titer in the shake flask batch cultures (**Fig. 2B**). Consequently, the specific protein productivity of the fed-batch culture for exponential growth phase (day 1 to 6) and for the whole process were calculated to be 11.7 pcd and 9.4 pcd respectively, 2.7 and 3.6 fold higher than the corresponding values of the shake flask batch culture. This improvement in protein productivities may be due to the lower specific growth rates of the cells, since these two parameters have been previously associated when temperature shift [49], [50] or sodium butyrate [51], [52] were applied to arrest cell growth for improved recombinant protein production.

While the recombinant protein titers achieved here are less than the grams per liter titers commonly reported for monoclonal antibodies (mAbs) and large recombinant proteins, it is comparable to reports of stable CHO cultures producing small recombinant proteins with molecular weight less than 50 kDa [53]–[56]. In addition, it is noteworthy that this titer has been attained using a stable cell pool, on which there are few published reports [31], [56]–[59]. From these reports, the titers achieved for small recombinant proteins range between 1.0 and 5.5 mg/l [31], [57], while that for mAbs or Fc-fusion proteins range between 5 and 1150 mg/l [56]–[59]. Hence, using our cell line development strategy, we have achieved the highest reported titer to our knowledge for producing small recombinant proteins less than 50 kDa using a stable cell pool.

### Purification and structural analysis of sDectin-1

**Figure 4:**
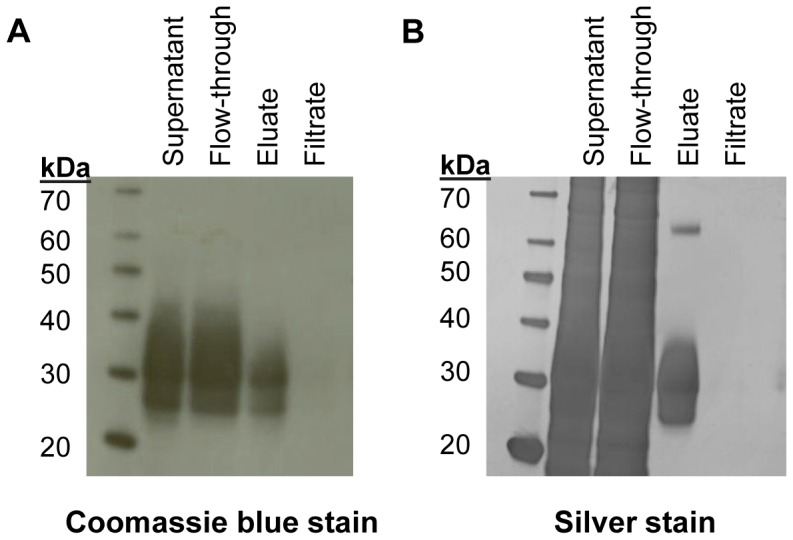
Purification of cHis-sDectin-1. cHis-sDectin-1 was purified using an IMAC nickel column and buffer exchanged using a 10 kDa molecular weight cut-off ultrafiltration spin filter. The unpurified supernatant, flow-through and eluate from the IMAC column, as well as the filtrate from the spin filter were separated by SDS-PAGE and stained using (A) Coomassie and (B) silver staining.

Recombinant cHis-sDectin-1 protein was purified from the culture supernatant with the use of an immobilized metal affinity chromatography (IMAC) nickel column which binds to the histidine tag on the C-terminal position of the protein. The purified proteins were concentrated and buffer exchanged into PBS using ultrafiltration spin columns, and subsequently quantified using a micro BCA (bicinchoninic acid) protein assay. The eluate from the IMAC nickel column was compared with the culture supernatant using Coomassie blue and silver-stained SDS-PAGE gel in **Fig. 4**. From the Coomassie blue-stained gel (**Fig. 4A**), we noted that the supernatant contains a major thick band that spans from 25 to 40 kDa. As this corresponds with the bands observed using Western blotting for mDectin-1 (**Fig. 2A**), it suggests that the recombinant cHis-sDectin-1 proteins are the major products in the protein-free culture medium. This is not surprising given the high titer of cHis-sDectin-1 achieved in the culture. In addition, we noted that the flow-through still contained a significant amount of sDectin-1. This was thus kept for further rounds of purification. The quality of the purification was further confirmed by the silver-stained gel (**Fig. 4B**), where a thick band similar to that observed from Western blotting (**Fig. 2A**) was observed for the eluate as compared to the smears for the lanes with the supernatant. This suggests that cHis-sDectin-1 protein of high purity was obtained. The filtrate from the ultrafiltration spin column showed no bands on both the Coomassie and silver-stained gels, suggesting that proteins were not lost from the filtering process.

**Figure 5:**
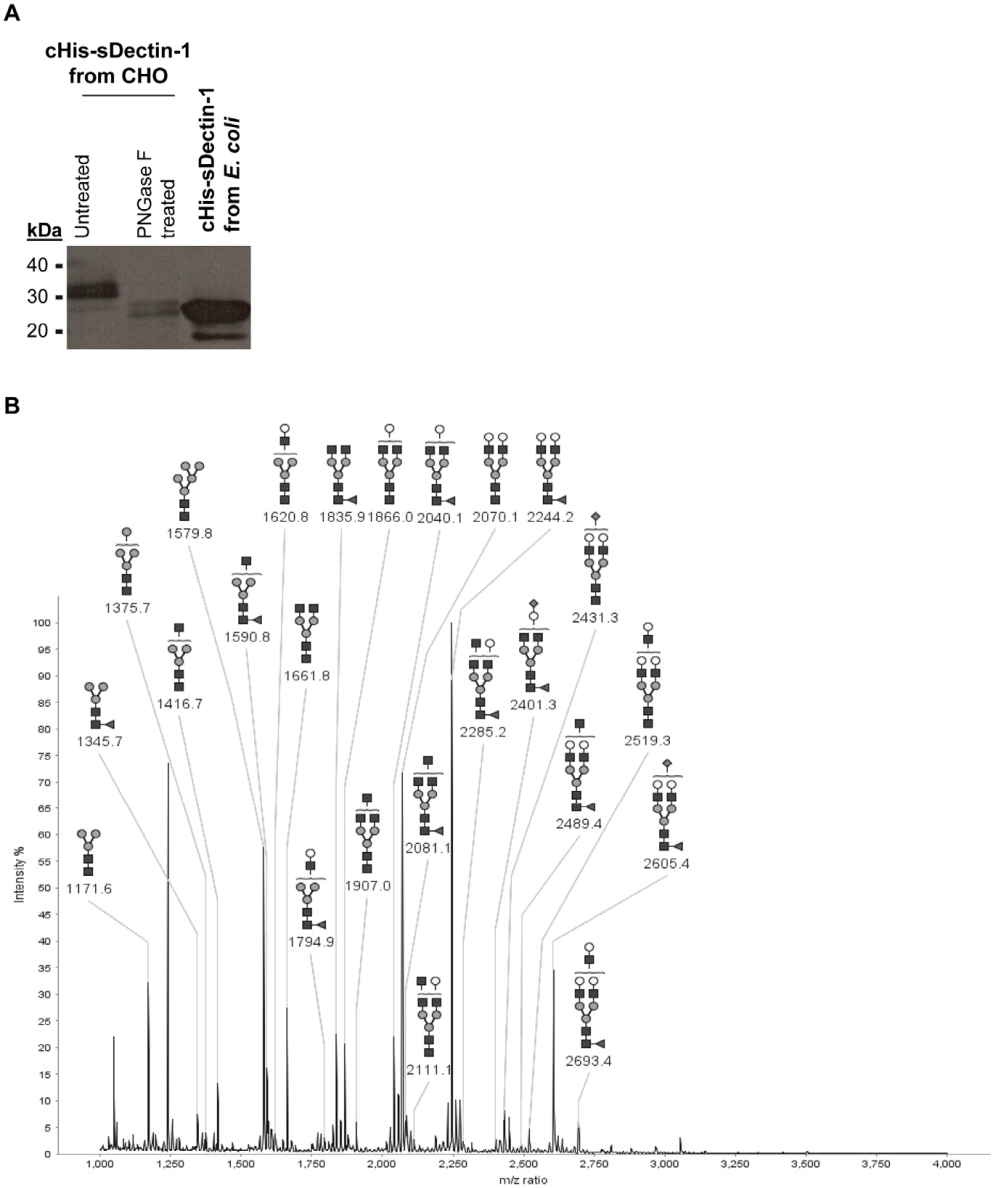
Structural analysis of cHis-sDectin-1. (A) Western blot analysis of untreated and PNGase F-treated cHis-sDectin-1 produced from CHO cell pool, compared to that produced by *E. coli*, using a horseradish peroxidase (HRP)-conjugated His-tag antibody (1∶ 2000; Catalog number 71840; Merck KGaA) (B) MALDI-TOF mass spectrometry analysis of the permethylated N-glycans released from the purified sDectin-1 produced from cHis CHO cell pool. Solid square, N-acetylglucosamine; solid circle, mannose; open circle, galactose; solid triangle, fucose; solid diamond, N-acetylneuraminic acid; open diamond, N-glycolylneuraminic acid.

Resolving the purified cHis-sDectin-1 protein further with Western blot, we observed that the recombinant protein has two main bands at 30 and 32 kDa, and a minor band at 25 kDa (**Fig. 5A**). This is in agreement with previous reports that described the presence of two major bands when mDectin-1 is expressed recombinantly [1], [22], although the differences between these major bands remained unclear at present. As the coding sequence translates into 21 kDa proteins based on the expected amino acid sequences (or a 23 kDa protein if the signal peptide is not cleaved), these recombinant proteins are likely glycosylated since 2 potential N-glycosylation sites are available (**Fig. 1B**). This was verified by removing the N-glycans from the cHis-sDectin-1 using a PNGase F digestion. Such treatment reduced the molecular weight of our recombinant cHis-sDectin-1 such that it was similar to the molecular weight of *E. coli* produced cHis-sDectin-1, confirming the presence of N-glycans on our recombinant cHis-sDectin-1 (**Fig. 5**). This observation is in agreement with previous reports describing the presence of glycans on full length mDectin-1 [1], [60]. Therefore, we decided to fully characterize the N-glycosylation profile of the purified cHis-sDectin-1 protein using a MALDI-TOF mass spectrometry approach. For this analysis, the cHis-sDectin-1 was digested into peptides and glycopeptides using trypsin digestion prior to a PNGase F treatment which released the N-glycans. Then, the free N-glycans were purified, permethylated and finally analyzed by MALDI-TOF mass spectrometry. The MALDI-TOF mass spectrum is presented in **Fig. 5B**. This showed a heterogeneous profile that contains mainly complex-type biantennary N-glycans with terminal galactose, with some glycans capped eventually with N-acetylneuraminic acid on the terminal position of their antennae. Although sDectin-1 may be glycosylated differently from endogenous mDectin-1, this analysis provided information on the possible N-glycosylation profiles of mDectin-1, which is difficult to analyze since it is a membrane receptor glycoprotein.

### Functional characterization of sDectin-1

**Figure 6:**
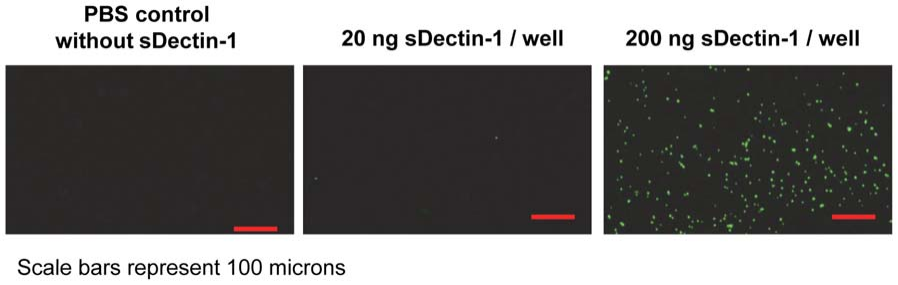
Binding of zymosan to sDectin-1. 20 or 200 ng cHis-sDectin-1 were coated on Maxisorp plates followed by FITC-zymosan. The plate was washed with PBS and imaged using a fluorescent microscope. Representative images from duplicate experiment are shown here.

As mDectin-1 is reported to bind β-glucans from yeast cell walls [1], [4]–[7], [18], [22], our cHis-sDectin-1 protein was tested for binding to zymosan particles which are mainly β-glucans. Briefly, purified cHis-sDectin-1 were coated onto high-binding 96 well plate, blocked with 3% BSA, incubated with FITC-zymosan, and washed with phosphate buffered saline. The wells were then viewed using a fluorescent microscope (**Fig. 6**). FITC-zymosan particles were observed to bind to wells coated with cHis-sDectin-1 in a concentration-dependent manner, but not at all in the blocked control well without the cHis-sDectin-1 coating. This demonstrates that our recombinant cHis-sDectin-1 is active and able to bind to zymosan, in agreement with previous reports.

**Figure 7:**
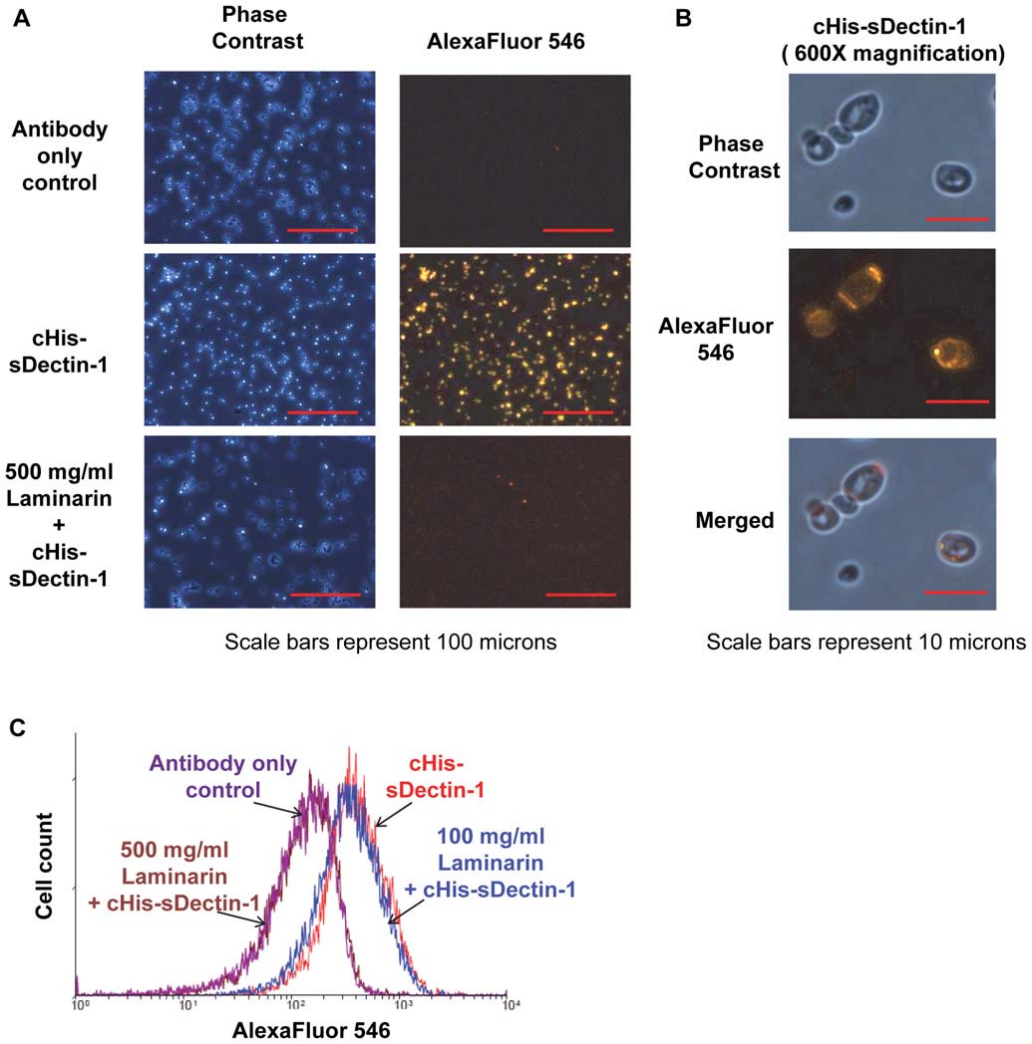
Binding of cHis-sDectin-1 to *Saccharomyces cerevisiae* cells. Purified cHis-sDectin-1 was diluted to a concentration of 25 µg/ml in blocking buffer with 500 µg/ml, 100 µg/ml or no laminarin. This was added to *Saccharomyces cerevisiae* yeast cells from overnight culture in blocking buffer (PBS with 3% FBS). The cells were then probed with an mDectin-1 goat polyclonal antibody (1∶200; AF1756; R&D Systems) and AlexaFluor546-conjugated anti-goat antibody (1∶100; Catalog Number A-11056; Molecular Probes). After which, the cells were washed with PBS and fixed using 4% paraformaldehyde. The cells were then resuspended in PBS and visualized by phase contrast and fluorescent microscopy at (A) 200× magnification and (B) 600× magnification. Images are cropped or scaled to fit the illustration. cHis-sDectin-1 stained yeast cells was also analyzed by flow cytometry (C).

To verify that our recombinant cHis-sDectin-1 also binds to yeast cell wall, immunofluorescence of cHis-sDectin-1 on *Saccharomyces cerevisiae* was performed (**Fig. 7**). The protein was observed to bind to the yeast cells, and this binding was blocked by competition with laminarin, a soluble β-glucan (**Fig. 7A**). This demonstrates that our recombinant cHis-sDectin-1 binds specifically to β-glucans present on yeast cell walls, similar to previous observations [4]–[7]. Under higher magnification, we observed that the binding of cHis-sDectin-1 was concentrated on some areas on the yeast cell surface (**Fig. 7B**). This corresponds to a previous report that showed the preferentially binding of another sDectin-1 to budding scars on *Saccharomyces cerevisiae* yeast [22]. The labeled yeast cells were also analyzed using flow cytometry to show similarly that recombinant cHis-sDectin-1 bound to yeast, and this binding was blocked by laminarin in a concentration dependent manner (**Fig. 7C**).

## Conclusions

Novel vectors utilizing an attenuated IRES element and a PEST-destabilized dhfr amplification marker have been constructed for an enhanced production of sDectin-1. Using these vectors and MTX amplification, we generated cell pools producing cHis- and nHis- sDectin-1 at titers of 246±27 mg/l and 158±57 mg/l respectively in shake flask batch cultures. The cell pool producing cHis-sDectin-1 was then scaled up using a 2 L stirred tank bioreactor in fed-batch culture to achieve a peak cHis-sDectin-1 titer of 598 mg/l and a specific protein productivity of 11.7 pcd at exponential growth phase. Using this approach, we have attained the highest reported titer to our knowledge for producing small recombinant proteins less than 50 kDa using a stable cell pool. Subsequently, cHis-sDectin-1 was purified using an IMAC nickel column. From the glycosylation analysis of the purified protein, we observed that cHis-sDectin-1 contains a heterogeneous profile of complex-type N-glycans. Functional analysis of cHis-sDectin-1 was also performed to demonstrate that our cHis-sDectin-1 binds to zymosan particles. Our cHis-sDectin-1 also binds to *Saccharomyces cerevisiae*, and this binding was blocked by laminarin, a soluble β-glucan. These demonstrate that our cHis-sDectin-1 is functional and binds to β-glucans.

## Materials and Methods

### Mice and ethics statement

Wild-type C57BL/6 mice were obtained from The Jackson Laboratory and bred in our facilities. Experiments on mice were performed according to guidelines from the National Advisory Committee on Laboratory Animal Research, with approval from the Institutional Animal Care and Use Committee (Permit number 110611). Mice were euthanized with CO_2_, and all efforts were made to minimize suffering.

### Construction of sDectin-1 expression vectors

Macrophages were differentiated from bone marrow of C57BL/6 mice as described previously [61]. Murine Dectin-1 gene was then cloned from first-strand cDNA of these bone-marrow derived macrophage cells and sequenced by Sanger sequencing. The sequence was then codon-optimized for CHO and synthesized (Genscript, Piscataway, NJ). DNA coding for the extracellular domain from amino acid 73 to 244 was obtained by polymerase chain reaction (PCR) from this sequence. Human IgG kappa light chain leader sequence, as well as 6× histidine tag, was then added to it via overlap PCR to form the sDectin-1 gene.

In parallel, the backbone for the sDectin-1 mammalian expression vector was constructed from pIRES (Clontech, Palo Alto, CA) by removing the f1 Ori and neomycin resistance cassette using restriction enzyme NaeI (New England Biolabs, Ipswich, MA). Destabilized dhfr ORF consisting of a chimeric dhfr-PEST sequence [1] was then cloned downstream of the IRES sequence using XbaI and NotI (New England Biolabs) restriction sites. Finally, the sDectin-1 gene was added to the vector using NheI and EcoRI restriction sites. (**Fig. S1**)

### Cell line and cell cultivation

Suspension CHO-DG44 cells (Gibco™ Catalog number 12609-012, Invitrogen, Carlsbad, CA) were previously adapted to a suspension medium (HyQ PF-CHO (Hyclone, Logan, UT) with 4 mM L-glutamine (Invitrogen) and 0.1% Pluronic® F-68 (Invitrogen)), that is supplemented with 0.1 mM sodium hypoxanthine and 16 µM thymidine (1×HT supplement, Invitrogen). Cells were routinely passaged every 3 to 4 days in 125 ml disposable Erlenmeyer flasks (Corning, Acton, MA). The cells were incubated on shaker platforms set at 110 rpm in a 37°C/8% CO2 humidified incubator. Cell densities and viabilities were determined by the Trypan Blue Exclusion method using an automated cell counter, Cedex (Innovatis, Malvern, PA), according to manufacturer's instructions.

Adherent CHO cells were cultivated in tissue culture flasks (T-flasks) during transfection and MTX amplification. DMEM media supplemented with 1% Fetal Bovine Serum (FBS) (Invitrogen) was used as culture medium, and the cells were incubated in a 37°C/5% CO2 humidified incubator. Accutase (PAA, Austria) was used to dislodge the cells for passaging.

### Generation of sDectin-1 producing cells

Prior to transfection, the expression vectors were linearized using BstBI (New England Biolabs), purified by ethanol precipitation and dissolved in sterile syringe-filtered 10 mM Tris–HCl, pH 8.5. For each transfection, 4 µg of purified vector was transfected into 1.5 million suspension CHO-DG44 cells using Cell Line Nucleofector® Kit V (Lonza Cologne, Germany) as per manufacturer's instructions. Transfected cells were transferred into 5 ml of prewarmed DMEM media supplemented with 1% Fetal Bovine Serum and 1× HT supplement (Invitrogen) in T25 flasks. After 48 hours, the medium was replaced with DMEM+1% FBS (selection medium) to start the selection process in the absence of HT. Selection medium was replaced every 5 days or when the medium turned yellow.

When the cells became confluent in selection medium, they were seeded into new T25 flasks in selection medium supplemented with 10 nM methotrexate (MTX) (Sigma, St. Louis, MO). Culture medium was changed every 5 days until the cells are confluent. The cells were passaged as such until they grew to confluency 5 days after passage. The process was then repeated at progressively higher MTX concentrations of 50 nM, 250 nM and 500 nM.

After the transfected cells recovered in selection medium with 500 nM MTX, they were seeded at 0.5×10^6^ cells per ml into a 500 nM MTX supplemented medium consisting of a 1∶1 mixture of selection medium and suspension medium. These were cultivated in 125 ml disposable Erlenmeyer flasks (Corning) on shaker platforms set at 110 rpm in a 37°C/8% CO2 humidified incubator. The cells were passaged at the same seeding density until culture viability was greater than 85%, upon which they were further adapted to suspension medium supplemented with 500 nM MTX.

### Sampling and characterization of shake flask cultures

Amplified cells were cultivated in 125 ml disposable Erlenmeyer flasks till their viabilities were greater than 85% before they were seeded into 30 ml medium at 0.5×10^6^ cells per ml in triplicates for cell growth and protein productivity characterization. Cell densities and viabilities were determined daily over a 7-day period by the Trypan Blue Exclusion method using an automated cell counter, Cedex (Innovatis). At each timepoint, 1 ml of culture sample was centrifuged at 5000 *g* for 10 minutes to pellet the cells. The culture supernatants were then transferred into clean tubes and stored at −80°C for further analysis. For protein purification, the culture was harvested on day 8 or when cell viability dropped below 50%.

Integral viable cell density (IVCD) was estimated by computing the area under the growth curve using Trapezium Rule. Specific protein productivities (q_p_) at exponential growth phase were determined as slopes of the straight trendlines from plots of sDectin-1 concentrations (determined using ELISA as described below) from days 1 to 3 against IVCD. Specific growth rates (μ) at exponential growth phase were determined as slopes of the straight trendlines from plots of log viable cell densities from days 1 to 3 against time.

### Bioreactor production of sDectin-1

The 500 nM MTX amplified cell pool producing cHis-sDectin-1 was seeded into a 2 L bioreactor containing 1.5 L HyQ PF-CHO medium (Hyclone) with 1 g/l glucose and 0.8 mM glutamine to obtain a viable cell density of 0.3×10^6^ cells/ml. The agitation rate, dissolved oxygen levels and pH of the culture were maintained at 115 rpm, 50% and 7.15 respectively. The culture set points for glucose and glutamine were 0.5 g/l and 0.3 mM respectively, and predictive feeding was used to maintain these concentration levels by the addition of a concentrated DMEM-based protein-free feed. From day 7, the feed was changed to a 200 g/l glucose solution to maintain only the glucose concentration in the culture. Cell counts and biochemical analyses were performed daily. Culture supernatant samples were also harvested daily and stored by short-term freezing at −20°C till further analysis. The culture was harvested on day 11 for protein purification. IVCD, q_p_ and μ were calculated as described above.

### Purification of sDectin-1

The harvested culture was centrifuged at 5000 *g* for 20 minutes to remove cells and debris. The recombinant protein was purified from the resultant supernatant by immobilized metal affinity chromatography (IMAC) using a 5 ml HisTrap FF nickel-sepharose column (GE Healthcare, United Kingdom) according to manufacturer's instructions. Concentration and diafiltration using Vivaspin 20 ultrafiltration spin columns with 10 kDa molecular weight cut-off (GE Healthcare) was then carried out on the eluted fractions. Protein purity was verified by silver staining as described below. Quantification was carried out using the Micro BCA Protein Assay Kit (Pierce, Rockford, IL).

### Gel electrophoresis analysis of sDectin-1

To separate the proteins on a gel, culture supernatant was mixed with NuPAGE® LDS Sample buffer (Invitrogen) and NuPAGE® Sample Reducing Agent (Invitrogen), and loaded onto a NuPage® Novex 4–12% Bis-Tris gel (Invitrogen) for electrophoresis. Following electrophoresis, proteins were visualized on the gel by Coomassie or silver staining, or transferred onto membranes for Western blotting, as described below.

For Coomassie staining, the gel was submerged in 0.1% Coomassie brilliant blue R250 for 1 hour and then destained in a 5% acetic acid/10% methanol solution until the protein bands can be visualized. For silver staining, the gel was first fixed in 50% methanol/5% acetic acid, and rehydrated with water. The gel was then sensitized with 0.02% sodium thiosulphate for 2 minutes and stained with 0.1% silver nitrate for 40 minutes at 4°C. Finally, the gel was developed using a 2% sodium carbonate/0.04% formaldehyde solution. For silver staining, the gel was rinsed twice with water for 1 minute between each step.

Western blotting was performed using the iBlot dry transfer system (Invitrogen) as per manufacturer's instructions. Histidine-tagged sDectin-1 was identified using horseradish peroxidase (HRP)-conjugated His-tag antibody (1∶ 2000; Catalog number 71840; Merck KGaA), or an mDectin-1 goat polyclonal antibody (1∶1000; AF1756; R&D Systems) with a HRP conjugated anti-goat antibody (1∶2000; Catalog number V8051; Promega, Madison, WI). Amersham ECL (GE Healthcare) was used to detect proteins.

### Verification of sDectin-1 production and signal peptide cleavage by peptide mass fingerprinting

In addition to Western blotting as described above, the production of recombinant sDectin-1 was verified by peptide mass fingerprinting. Samples of culture supernatant were first separated on an SDS-PAGE gel as described above. The corresponding bands on the gel were excised and soaked overnight in washing solution (25 mM ammonium bicarbonate in 50% acetonitrile/water). The solution was replaced with acetonitrile and incubated for 10 min at room temperature. The gels were then dried in a Savant Speedvac concentrator (Thermo Scientific, Waltham, MA). The dried gel was incubated at 56°C for 1 h in 10 mM DTT. The solution was then replaced with 55 mM iodoacetamide and incubated at room temperature for 45 min in the dark. The gel was further washed with 100 mM ammonium bicarbonate for 10 min, and dehydrated in 100% acetonitrile and incubated for 10 min. The gels were dried in a Savant Speedvac concentrator again, prior to overnight trypsinization (Promega, Madison, WI) at 37°C according to manufacturer's instructions. Peptides were extracted with 50% acetonitrile/0.1% TFA for 5 min and concentrated using the Savant Speedvac. The digested peptides were added to 2% formic acid/1% methanol loading buffer, prior to LC-MS/MS (*nano*Acquity UPLC coupled to Synapt G2 HDMS system; Waters, Milford, MA) analysis. The raw mass spectrometry data were processed using the ProteinLynx Global Server software (Waters) at false discovery rate of 1%. The digested peptides were also analyzed by MALDI-TOF mass spectrometry (Absciex).

### ELISA quantification of sDectin-1 concentration in culture supernatant

96-well maxisorp plates (Nunc) were coated overnight at 4°C with an mDectin-1 monoclonal antibody (1∶500; MAB 1756; R&D Systems, Minneapolis, MN) diluted in a sodium bicarbonate coating buffer (pH 8.0). Blocking was performed by incubating with Superblock (Pierce) for three times of 5 minutes each on a shaker platform. 100 µL of appropriately diluted samples were then added to the wells for 1 hour. The plates were subsequently incubated with horseradish peroxidase (HRP)-conjugated His-tag antibody (1∶ 2000; Catalog number 71840; Merck KGaA, Germany) for 1 hour. Subsequently, 100 µL of *o*-Phenylenediamine dihydrochloride (Sigma) was added for 30 minutes at 37°C; following which the reaction was quenched with 100 µL of 3M HCl and absorbance measured at 492 nm using a reference wavelength of 620 nm. Washing using PBS with 0.1% Tween-20 for three times of 5 minutes each was carried out after each incubation step. Quantification of sDectin-1 concentrations was performed by comparing the samples' absorbance against a standard curve generated using cHis- or nHis- sDectin-1 that were purified and quantified as described above.

### Glycosylation analysis of sDectin-1

200 µg of purified cHis-sDectin-1 were digested in a 50 mM ammonium bicarbonate buffer pH 8.2 with 8 µg of trypsin (Sequencing grade modified trypsin, Promega Corporation, USA) at 37°C for 4 hours. The digestion was then quenched by heating the sample at 95°C for 15 minutes. The resulting mixture was deglycosylated by incubation with 70 U of PNGase F (Prozyme, USA), overnight at 37°C. A C_18_ Sep-Pak® cartridge (Waters) was then used to separate released N-glycans from peptide mixtures. Briefly, the C_18_ Sep-Pak® cartridge was primed sequentially with 5 ml of respectively methanol, deionized water, acetonitrile and deionized water before loading the sample onto the cartridge. Released N-glycans were washed out using 5 ml of deionised water and 5 ml of acetic acid 5%. The fractions were dried down prior to permethylation.

For the permethylation, sodium hydroxide pellets were first crushed using an agate mortar in dimethyl sulfoxide. 500 µl of the sodium hydroxide slurry and 500 µl of iodomethane were then added to resuspend the dried glycan samples in glass tubes. The reaction proceeded under rotation at 20 rpm for 2 h. 1 ml of deionised water was added drop-wise to quench the permethylation reaction. The permethylated samples were then extracted with 2 ml of chloroform and washed several times with deionized water until the chloroform layer was clear. The chloroform layer was then evaporated to dryness.

The dried permethylated N-glycan samples were reconstituted in 200 µl of 50% methanol and loaded onto the Sep-Pak® cartridges. Elution was performed using 2 ml of 15, 35, 50 and 75% acetonitrile respectively for each sample. Each eluted fraction was then collected and evaporated to dryness using vacuum centrifuge prior to mass spectrometry analysis.

MALDI-TOF data was acquired with the 5800 MALDI-TOF/TOF mass spectrometer (AB Sciex, Foster City, CA) in positive reflectron mode as previously described [46]. Both the 35% and 50% acetonitrile fractions were observed to contain permethylated N-glycans.

### Functional characterization of sDectin-1

To assay for binding to zymosan particles, FITC-zymosan was prepared by diluting 1 µl of FITC-zymosan particles (Invitrogen) in 3.0 ml of blocking buffer. This mixture was sonicated for 1 minute on ice to disperse the zymosan particles. 96-well Maxisorp plates (Nunc) were coated overnight at 4°C with 20 or 200 ng purified sDectin-1 diluted in PBS (pH 7). The wells were washed once using 200 µl of blocking buffer (PBS+3% BSA) and blocking was performed by incubating the plate with blocking buffer for 1 hour at room temperature. 100 µl of the diluted zymosan mixture was then added and the plate was incubated for 1 hour at room temperature. The wells were subsequently washed 2 times using 200 µl of PBS and viewed using a fluorescent microscope.

To characterize binding to *Saccharomyces cerevisiae*, purified sDectin-1 was diluted in blocking buffer to a concentration of 25 µg/ml. For some samples, laminarin (Sigma) was added to the blocking buffer used to dilute the sDectin-1 to test the binding specificity. The diluted sDectin-1 was incubated on ice for 1 h. *Saccharomyces cerevisiae* yeast was cultivated overnight by inoculating into sterile YPD medium (1% Bacto yeast extract (BD, Sparks, MD), 2% Bacto peptone (BD), 2% D-glucose (Sigma)) and incubating overnight on shaker platforms set at 180 rpm in a 28°C humidified incubator. The yeast culture was then centrifuged at 2000 *g* for 5 minutes, resuspended in blocking buffer (PBS with 3% FBS), and incubated on ice for 30 minutes. Equal volumes of the yeast suspension were aliquoted into different eppendorf tubes. The cells were then resuspended with diluted sDectin-1 and incubated on ice for 1 hour. After which, the cells were probed with an mDectin-1 goat polyclonal antibody (1∶200; AF1756; R&D Systems) and AlexaFluor546-conjugated anti-goat antibody (1∶100; Catalog Number A-11056; Molecular Probes, Eugene, OR). The cells were washed twice with cold blocking buffer between each incubation with the different reagents. Finally, the cells were washed with PBS and fixed using 4% paraformaldehyde (BDH Lab Supplies, England) in PBS at room temperature for 30 min. After fixing, the cells were resuspended in PBS and analyzed using fluorescence microscopy and flow cytometry (FACScalibur, BD Biosciences, Mountain View, CA).

## Supporting Information

Figure S1 **Construction of sDectin-1 expression vectors.** The f1 Ori and neomycin resistance cassette was first removed from pIRES using restriction enzyme NaeI. The chimeric dhfr-PEST sequence was then inserted downstream of the IRES sequence using XbaI and NotI restriction sites. The sDectin-1 gene, consisting of a secretion signal peptide, a 6× histidine tag and mDectin-1 ECD, was added upstream of the IRES using NheI and EcoRI restriction sites.
(TIF)
