# Supplementary figures and images for: Production of Functional Soluble Dectin-1 Glycoprotein Using an IRES-Linked Destabilized-Dihydrofolate Reductase Expression Vector
Source: PLoS One. 2012 Dec 26;7(12):e52785. doi: 10.1371/journal.pone.0052785 (PMC3530475; doi:10.1371/journal.pone.0052785)

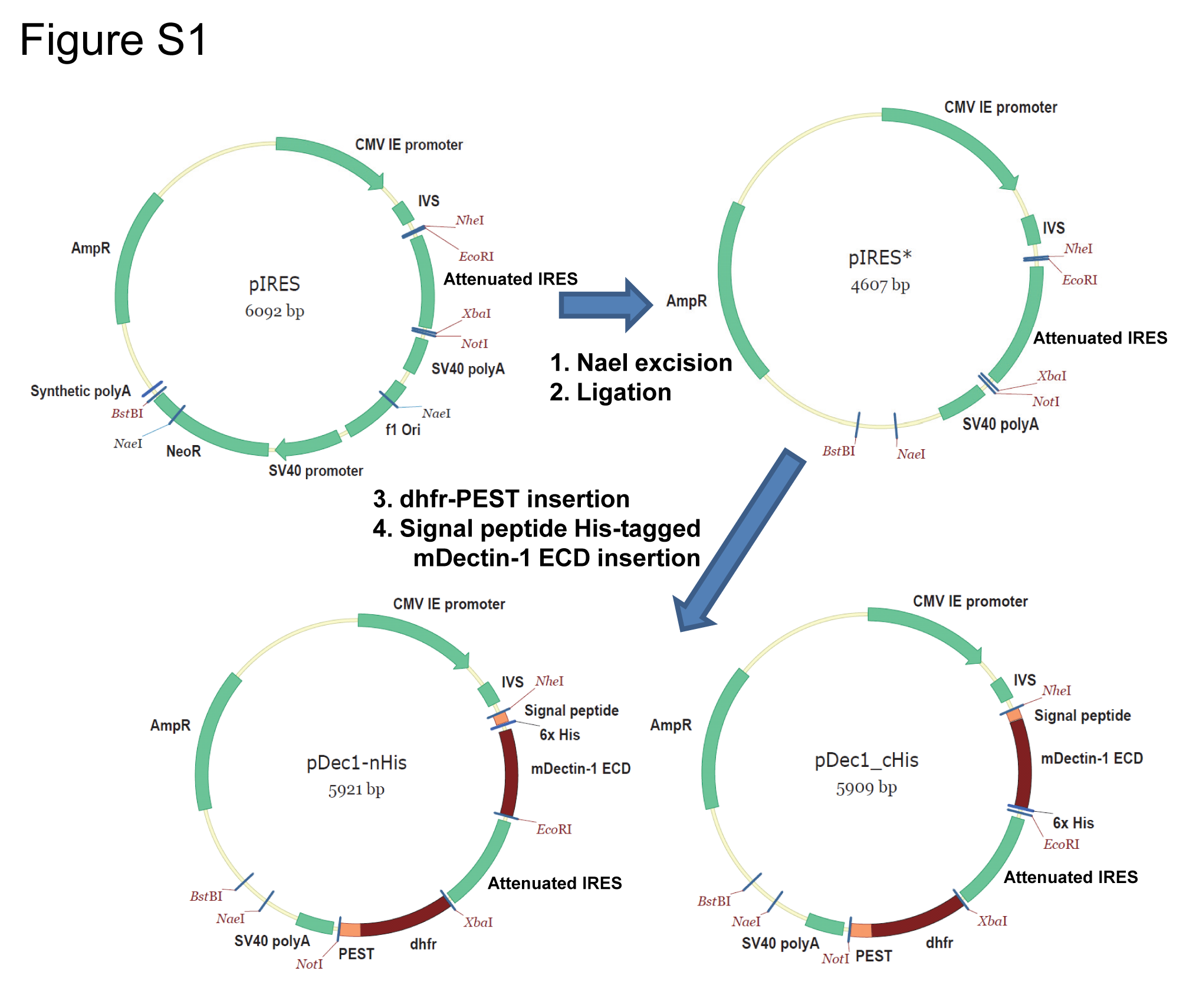

Supplement: Figure S1 — Construction of sDectin-1 expression vectors. The f1 Ori and neomycin resistance cassette was first removed from pIRES using restriction enzyme NaeI. The chimeric dhfr-PEST sequence was then inserted downstream of the IRES sequence using XbaI and NotI restriction sites. The sDectin-1 gene, consisting of a secretion signal peptide, a 6× histidine tag and mDectin-1 ECD, was added upstream of the IRES using NheI and EcoRI restriction sites. (TIF) [file pone.0052785.s001.tif]
